# What to advise to patients with only one good quality blastocyst, PGT-A or not? Outcomes of 2064 cycles

**DOI:** 10.1007/s10815-022-02617-7

**Published:** 2022-09-20

**Authors:** Semra Kahraman, Ipek Nur Balin Duzguner, Yucel Sahin, Tulay Irez

**Affiliations:** 1 ART and Reproductive Genetics Center, Istanbul Memorial Hospital, Piyalepasa Bulvari, Sisli, 34384 Istanbul, Turkey; 2 Faculty of Medicine, Department of Histology and Embryology, Biruni University, Istanbul, Turkey

**Keywords:** Single blastocyst, PGT-A, Clinical pregnancy, Live birth

## Abstract

**Purpose:**

To evaluate whether preimplantation genetic testing for aneuploidy (PGT-A) is beneficial for patients who have only one blastocyst available for biopsy or transfer.

**Methods:**

This retrospective study was based on 1126 single blastocyst PGT-A and 938 non-PGT-A cycles, a total of 2064 ART cycles which resulted in a single good quality blastocyst in women between 20 and 45 years old. The PGT-A group had 225 single euploid embryo transfer cycles and the non-PGT-A group had 938 single blastocyst embryo transfer cycles.

**Results:**

In the generalized linear mixed model (GLMM), female age and PGT-A variables were found to be significant variables on pregnancy outcomes. In the PGT-A cases, regardless of the effect of other variables, the probabilities of clinical pregnancy and live birth were found to be 3.907 and 3.448 fold higher respectively than in the non-PGT-A cases (*p* < 0.001). In non PGT-A cases, the probability of a total pregnancy loss was found to be 1.943 fold higher (*p* = 0.013).

**Conclusion:**

PGT-A in the presence of a single blastocyst significantly increases clinical pregnancy and live birth rates and decreases total pregnancy losses regardless of age. In addition, aneuploid embryo transfer cancelations prevent ineffective and potentially risky transfers.

## Introduction

It is well known from previous studies that aneuploidy rate increases with advanced maternal age [1–3]. Preimplantation genetic testing for aneuploidy (PGT-A) has been shown to reduce the risk of miscarriage particularly in advanced maternal age patients ≥ 35 years old [1, 4]. Especially in the presence of a large number of embryos suitable for biopsy, PGT-A shortens the time to reach a healthy pregnancy by selecting chromosomally normal embryos with a high chance of implantation and low risk of miscarriage [5, 6]. Finding at least one transferable euploid embryo is the main determinant of clinical success in PGT-A. Studies have shown that the higher the number of blastocysts to be biopsied, the higher the chance of finding at least one euploid embryo. When controlled for age, the probability of finding at least one euploid embryo increases significantly with each additional oocyte retrieved [7–10]. However, is PGT-A a good option for patients with low ovarian reserve who do not have multiple embryos for biopsy? Our study investigates this question, aiming to obtain the information necessary to be able to advise patients with only one good quality blastocyst whether or not to undergo PGT-A.

## Material and methods

### Design

This retrospective single center study was conducted at Istanbul Memorial Hospital, ART and Reproductive Genetics Center. Data was obtained from our Aura database. Our study group, the vast majority of whom had diminished ovarian reserve (DOR), consisted of cases with any of the following: advanced maternal age, a history of recurrent spontaneous abortion (RSA), a history of repeated implantation failure (RIF), a known pregnancy termination due to a chromosomally abnormal fetus or a combination of two or more of these factors. A total of 2064 assisted reproductive technology (ART) cycles performed between August 2011 and March 2021 in women between 20 (min) and 45 (max) years old and which resulted in a single, transferable, good, or top-quality blastocyst were analyzed. In the PGT-A group, as there was only one available euploid embryo for transfer, some moderate quality embryos (1.7%) and some delayed re-expanded embryos (0.8%) had to be transferred. A total of 1126 cycles were PGT-A cycles, while the non-PGT-A group comprised 938 non-PGT-A cycles. The vast majority of the non-PGT-A group had DOR (73%) and only one embryo available for transfer. Infertility factors in PGT-A and non-PGT-A groups are shown in Table 1.

**Table 1:** Patient demographics, infertility factors, and ART cycle characteristics of patients that had only a single blastocyst with PGT-A or without PGT-A

	Single blastocyst with PGT-A (n:1126)	Single blastocyst without PGT-A (n:938)	*P*-value
Age (years)	38.61 ± 0.13	35.26 ± 0.14	< 0.001*
BMI (kg/m^2^)	25.27 ± 0.12	24.99 ± 0.13	0.025*
Duration of infertility (years, *n*)	5.43 ± 0.14	5.32 ± 0.15	**0.531**
History of recurrent spontaneous abortion, %	18.7%	6.7%	< 0.001*
History of recurrent implantation failure, %	25.6%	14.3%	< 0.001*
Unexplained infertility, *n* (%)	23 (2%)	74 (7.8%)	< 0.001*
Male factor, *n* (%)	420 (37.3%)	407 (43%)	0.06
Tubal factor, *n* (%)	119 (9.7%)	87 (9.2%)	0.768
Endometriosis, *n* (%)	188 (15.3%)	150 (15.9%)	0.720
Diminished ovarian reserve, *n* (%)	964 (85.6)	685 (73%)	< 0.001*
Advanced maternal age, *n* (%)	981(86.9%)	293(25.8%)	< 0.001*
AMH (ng/mL)	1.11 ± 0.04	1.26 ± 0.04	0.001*
Total gonadotropin dosage used (IU)	2210.32 ± 38.13	2081.12 ± 40.47	0.018*
Estradiol level on trigger day (ng/L)	994.18 ± 27.99	1179.36 ± 29.95	< 0.001*
Number of aspirated oocytes, *n*	4.44 ± 0.14	5.76 ± 0.14	< 0.001*
MII, *n*	3.58 ± 0.11	4.51 ± 0.11	< 0.001*
PN2, *n*	2.66 ± 0.08	3.35 ± 0.08	< 0.001*
Maturation rate, %	87.49 ± 0.58	83.74 ± 0.63	< 0.001*
Fertilization rate, %	82.40 ± 0.75	80.20 ± 0.80	0.045*

*BMI* body mass index, *AMH* anti-mullerian hormone, *MII* mature oocytes, *PN2* normally fertilized oocytes, *PGT-A* preimplantation genetic testing-aneuploidy. *p < 0.05

Patients who underwent PGT for the indication of a known chromosomal translocation or single-gene disorders were not included in the study. Mosaic embryo transfers were also excluded from this study, firstly, because in 21.7% of cases aCGH was used, which may not detect mosaicism. Secondly, precise matching of mosaicism in both PGT-A and non-PGT-A groups would not have been feasible since the percentage of mosaicism and the number of chromosomes involved would vary widely in their incidence and effect on pregnancy outcomes.

This study was approved by Istanbul Memorial Hospital institutional review board (Number:01042022/002).

In 1126 PGT-A cycles, 1126 embryos were biopsied. In 225 (20%) out of 1126 PGT-A cycles a transferable, chromosomally normal embryo was found.

In these cases, modified natural frozen embryo transfer (mNC-FET) (*n* = 126, 56%) or artificial frozen embryo transfer (ERT-FET) (*n* = 99, 44%) cycles were performed. In the non-PGT-A group (*n* = 938 cycles), the majority of patients received fresh embryo transfer (*n* = 845), whereas 93 cases had frozen embryo transfer, either mNC-FET (*n* = 46, 49.5%) or ERT-FET (*n* = 47, 50.5%). In the non-PGT-A group, the reasons for freezing the single blastocysts were either social factors or medical factors such as hysteroscopy for endometrial polyps or submucosal myoma, or thin endometrium (< 7 mm), or laparoscopy for myoma uteri or hydrosalpinx.

In our study, DOR was defined in accordance with the Bologna ESHRE criteria, in which at least two of the following are present: (1) abnormal ovarian reserve tests with antimullerian hormone (AMH) < 1.1 ng/ml or antral follicle count (AFC) < 5–7 follicles, (2) previous poor ovarian response, or (3) advanced maternal age (≥ 40 years) or other risk factor for DOR (11).

In 244 cycles (21.7%), array comparative genomic hybridization (aCGH) was performed for trophoectoderm biopsy testing. Next generation sequencing (NGS) was used in 882 out of 1126 PGT-A cycles (78.3%).

Demographic parameters, cycle characteristics, and pregnancy outcomes were compared between PGT-A and non-PGT-A cycles. Univariable and multivariable evaluations of the patients and cycle characteristics in relation to clinical pregnancy, total pregnancy loss, and live birth were done.

### Ovarian stimulation

For controlled ovarian stimulation (COS), in the vast majority of the patients (*n* = 2043), GnRH antagonist protocols were used and in a minority of the patients (*n* = 21), gonadotropin-releasing hormone (GnRH) analog supression protocols were used. Recombinant follicle stimulating hormone (rFSH) (Gonal-f; Merck, Switzerland) or a combination of rFSH and recombinant luteinizing hormone (rLH) (Luveris; Merck, Switzerland) or human menopausal gonadotropin (hMG) (hMG, Ferring, Switzerland) were used to stimulate ovaries with initial doses based on the individual characteristics of patients. Oocyte retrievals were carried out by transvaginal ultrasound guidance 36 h after the injection of 250 mcg recombinant human chorionic gonadotropin (rhCG) (Ovitrelle; Merck, Switzerland) or GnRH analog (Lucrin; Abbott Laboratories, USA) by transvaginal ultrasound guidance.

### Embryo scoring

IVF culture media, a single-step medium for uninterrupted embryo culture (LifeGlobal, Cooper Surgical, Brussels, Belgium) and incubation system, were the same for all cases. Blastocysts were scored before vitrification according to Gardner’s classification and classified into three groups: excellent quality (TQ), good quality (GQ), moderate and poor quality (PQ) blastocysts. The TQ designation includes 3AA, 4AA, and 5AA blastocysts, whereas GQ comprises those graded as 3/4/5BB, AB, or BA. Blastocysts of moderate and inferior quality were designated as PQ blastocysts. Moderate quality was defined as 2AA, 2AB, 2 BA blastocysts, and at least 95% vitality. Poor quality was defined as 2BB, 2CC,3/4/5 BC, 3/4/5 CB, morula, or below 90% vitality for all blastocysts [12].

### Trophectoderm biopsy

Trophectoderm biopsy involved making a hole in the zona pellucida by using diode laser (RI Saturn 3, England) on day 3 of embryonic development, which allowed the developing trophectoderm cells to protrude after blastulation, facilitating the biopsy. On day 5 or 6 after fertilization, between five and eight cells were excised using laser energy, without loss of inner cell mass. If the embryo was on day 6 or if the hatching part of the embryo had excessive trophectoderm cells, both laser and mechanic techniques were used. A mechanical cut was performed using a pipette with a 30 mm inner diameter (Origio, Denmark).

If a small number of cells protruded or if the trophectoderm score was B, then detachment was done by mechanical cutting only.

Two PGT-A techniques were used to study biopsy material: NGS and in a minority of cases aCGH. aCGH was performed between 2011 and 2016 using 24Sure kit (Illumina, USA) following standard procedures in the provided manual. Analyses were done using BlueFuse Multi Analysis Software (Illumina, USA), illustrating the chromosome copy numbers in a biopsy sample. NGS was performed between 2017 and 2020 using ReproSeq kit (ThermoFisher, USA) and initially PGM (Ion Personal Genome Machine, ThermoFisher, USA) and latterly S5 (ThermoFisher, USA). Analyses were performed on Ion Reporter software suite v5.2 and v5.6 (ThermoFisher, USA).

### Embryo vitrification and thawing

Good or top-quality blastocysts (at least 3BB) were vitrified on the morning of day 5 or the morning of day 6 with Kitazato vitrification media, using cryotops® as carriers. In this DOR patient group with only one embryo, to allow for the possibility, no matter how slight, of a slow growing embryo developing to blastocyst stage, incubation was continued to day 5 (114–120 h post-ICSI) or even day 6 (135–144hpi). Blastocysts were thawed with Kitazato warming media according to the manufacturer’s instructions. Embryos were first checked for survival 30 min after thawing. Two hours after warming, a second check was performed for re-expansion, hatching, extensive cytoplasmic granulation, and the presence of necrotic foci, which are predictors of the rates of implantation, pregnancy, and live birth [13]. Eligible blastocysts with at least 80% re-expansion and vitality were transferred in the afternoon of the same day. In neither group was there any case of failure to re-expand. There was a very small number of cases of delayed re-expansion (up to 4 h): in the PGT-A group, only 0.8% and in non-PGT-A group, 4.2%.

### Endometrial preparation for frozen embryo transfer cycles

All patients undergoing mNC-FET and ERT-FET were examined on the second day of the menstrual period by transvaginal ultrasonography to rule out the presence of ovarian cysts or other pelvic pathologies. In mNC-FET cycles, a second transvaginal ultrasonography was performed 7–9 days after the first examination according to the menstrual cycle length to observe whether there was a spontaneously growing dominant follicle. When the dominant follicle reached 15 mm, serum estradiol and LH levels were monitored every 24 h until LH surge was detected. When the endometrial thickness reached above 8 mm and the LH level rose above, a critical threshold level (> 15 IU/L), r-hCG (Ovitrelle, Merck-Serono, Switzerland) was administered [14]. Frozen embryo transfer was performed 6 days after rhCG administration. In ERT-FET cycles, transvaginal ultrasonography was repeated on day 10 or 11 and 14 or 15, to observe the endometrial thickness and whether there was a spontaneously growing dominant follicle or cyst. When the endometrial thickness reached above 8 mm and there was no spontaneously growing follicle, frozen embryo transfer was scheduled for 5 days after the start of luteal phase support.

### Luteal phase support

For luteal phase support, in mNC-FET cycles vaginal progesterone gel, 90 mg (8%) (Crinone® Merck Serono, Switzerland) was administered once a day starting 2 days after rhCG administration. In ERT-FET cycles, vaginal progesterone gel 90 mg (8%) (Crinone® Merck Serono, Switzerland) was administered twice a day, beginning on the 15th day of menstruation at the earliest and when the endometrium reached 8 mm. In fresh embryo transfer cycles, vaginal progesterone gel 90 mg (8%) (Crinone® Merck Serono, Switzerland) was administered twice a day, starting the day after oocyte retrieval.

Nine days after blastocyst transfer, serum β-hCG was measured. When pregnancy occurred, the same daily doses of progesterone were continued until the 12th week of gestation. At 7 weeks, a transvaginal ultrasound was performed to monitor early pregnancy. A viable pregnancy was defined as the presence of fetal heartbeat at 7 weeks.

### Statistical analysis

Number Cruncher Statistical System (NCSS) Statistical Software (Utah, USA) program was used for the statistical analyses. During the evaluation of the data obtained from the study, the Shapiro Wilk test and graphs were used regarding the conformity of the descriptive statistical methods (mean, standard deviation) as well as the variables to a normal distribution. Pearson’s chi-square test and Fisher’s exact test were used for comparison of categorical variables.

Generalized linear mix model (GLMM) was used for univariate and multivariate evaluations of risk factors affecting clinical pregnancy, total pregnancy loss, and live birth.

## Results

Patient demographics, infertility factors, and ART cycle characteristics of patients that had only a single blastocyst with PGT-A or without PGT-A are shown in Table 1.

The number of patients diagnosed with unexplained infertility was significantly lower in the PGT-A group compared to the non-PGT-A group (*p* < 0.001). On the other hand, the numbers of patients with advanced maternal age and DOR were significantly higher in PGT-A cases (*p* < 0.001). There was no statistically significant difference in the percentages of male factor, tubal factor, and endometriosis between the two groups.

The mean female age, history of RSA, and history of RIF and DOR were significantly higher in the PGT-A group (*p* < 0.001). AMH level and estradiol level on trigger day were significantly lower whereas the total gonadotropin dosage used was significantly higher in the PGT-A group (*p* < 0.001). The mean number of aspirated, MII and fertilized oocytes were significantly lower in the PGT-A group (*p* < 0.001).

Although the PGT-A group included older patients with a lower ovarian reserve and even though the number of aspirated, mature, and fertilized oocytes were lower than in the non-PGT-A group, after consultation regarding its possible advantages and disadvantages, PGT-A was recommended because of a multiple number of unsuccessful previous cycles, a history of RSA and RIF.

Oocyte maturation rate was calculated by dividing the number of mature oocytes by the number of oocytes retrieved. Fertilization rate was calculated by dividing the number of fertilized oocytes by the number of mature oocytes.

Table 2 shows the comparison of pregnancy outcomes of patients with a single blastocyst who underwent embryo transfer with or without PGT-A. The implantation rate was calculated by dividing the number of gestational sacs by the number of embryos transferred. The implantation rate was significantly higher (63%) in the PGT-A group compared to the non-PGT-A group (39.3%) (*p* < 0.001). Biochemical pregnancy and clinical pregnancy rates per embryo transfer were significantly higher in the PGT-A group (71% and 63% respectively) compared to the non-PGT-A group (44.8% and 38.6% respectively) (*p* < 0.001). Total pregnancy loss was found to be lower in PGT-A group than non-PGT-A group; however, this was not significant (*p* = 0.49). Live birth rate per embryo transfer was significantly higher in PGT-A group compared to non-PGT-A group (51.1%, 29.6%; *p* < 0.001).

**Table 2:** Comparison of pregnancy outcomes of patients with a single blastocyst who underwent embryo transfer with and without PGT-A

	PGT-A (euploid ET) cycles (n:225)	Non PGT-A ET cycles (n:938)	*P*-value
Biochemical pregnancy/ET, *n* (%)	158/225 (71%)	420/938 (44.8%)	< 0.001*
Clinical pregnancy/ET, *n* (%)	131/225 (63%)	362/938 (38.6%)	< 0.001*
Implantation rate, *n* (%)	131/225 (63%)	369/938 (39.3%)	< 0.001*
Total pregnancy losses, *n* (%)	40/158 (25.3%)	131/420 (31.2%)	0.493
Live birth/ET, *n* (%)	115/225 (51.1%)	278/938 (29.6%)	< 0.001*

^*^p < 0.05. *ET* embryo transfer

In the univariable analysis, female age, DOR, AMH, and PGT-A variables had a significant effect on clinical pregnancy (Table 3). Variables with a significant effect in univariable evaluations were included in multivariable analysis as independent variables. In the GLMM, female age and PGT-A were found to be significant variables. The probability of a clinical pregnancy was 5.548 fold higher in cases under 35 years of age (*OR* (%95 *CI*) = 5.548 (2.198, 14.002), *p* < 0.001). The probability of a clinical pregnancy was 4.497 fold higher in cases between the ages of 35–38 (*OR* (95% *CI*) = 4.497 (1.77, 11.429), *p* = 0.002). In PGT-A cases, the probability of a clinical pregnancy was found to be 3.907 fold higher (*OR* (95% *CI*) = 3.907 (2.742, 5.567), *p* < 0.001).

**Table 3:** Results of univariate and multivariate factors in relation to the clinical pregnancy

Clinical pregnancy	Univariable		Multivariable	
	*OR* (95% *CI*)	*P*-value	*OR* (95% *CI*)	*P*-value
Age group (years)		< 0.001*		< 0.001*
< 35	5.229 (2.147, 12.731)	< 0.001*	5.548 (2.198, 14.002)	< 0.001*
35–38	4.721 (1.912, 11.655)	0.001*	4.497 (1.77, 11.429)	0.002*
39–40	3.239 (1.267, 8.283)	0.014*	2.291 (0.863, 6.08)	0.096
41–43	2.701 (1.042, 7.003)	0.041*	2.042 (0.76, 5.491)	0.157
> 43	Reference	-	Reference	-
BMI (kg/m2)	1.005 (0.979, 1.031)	0.720		
Duration of infertility (years, *n*)	0.989 (0.963, 1.016)	0.415		
RSA (*n*)	1.01 (0.677, 1.506)	0.962		
DOR (*n*)	0.74 (0.564, 0.971)	0.030*	1.048 (0.698, 1.573)	0.820
AMH (ng/ml)	1.083 (1.003, 1.169)	0.042*	1.046 (0.944, 1.159)	0.394
Totaly gonadotropin dosage used (IU)	1 [1, 1]	0.285		
Estradiol level on trigger day (ng/L)	1 (1, 1)	0.179		
Number of aspirated oocytes (*n*)	1.021 (0.998, 1.045)	0.071		
PGT-A (*n*)	2.716 (1.982, 3.722)	< 0.001*	3.907 (2.742, 5.567)	< 0.001*

*BMI* body mass index, *RSA* recurrent spontaneous abortion, *DOR* diminished ovarian reserve, *AMH* Anti-Mullerian hormone, *PGT-A* preimplantation genetic testing-aneuploidy. *GLMM* generalized linear mixed model, *OR* odds ratio, *CI* confidence interval. *p < 0.05

In the univariable analysis, no variable was found to have a significant effect on total pregnancy loss (Table 4). PGT-A and age variables were included in multivariable analysis as independent variables, even though they did not have significant effects in univariable analysis. In the GLMM, female age and non-PGT-A variables were found to be significant variables. The probability of a total pregnancy loss was 2.712 fold higher in cases between 41 and 43 years (*OR* (%95 *CI*) = 2.712 (1.32, 5.57), *p* = 0.007). In non PGT-A cases, the probability of a total pregnancy loss was found to be 1.943 fold higher (*OR* (%95 *CI*) = 1.943 (1.148, 3.289), *p* = 0.013).

**Table 4:** Results of univariate and multivariate factors in relation to the total pregnancy loss

Total pregnancy loss	Univariable		Multivariable	
	*OR* (95% *CI*)	*P*-value	*OR* (95% *CI*)	*P*-value
Age group (years)		0.102		0.016*
< 35	Reference	-	Reference	-
35–38	1.788 (0.386, 8.287)	0.457	0.82 (0.517, 1.3)	0.397
39–40	1.834 (0.967, 3.479)	0.063	1.817 (0.92, 3.588)	0.085
41–43	1.223 (0.672, 2.226)	0.509	2.712 (1.32, 5.57)	0.007*
> 43	0.734 (0.467, 1.153)	0.179	2.466 (0.511, 11.902)	0.261
BMI (kg/m2)	1.023 (0.984, 1.064)	0.243		
Duration of infertility (years, *n*)	1.003 (0.963, 1.045)	0.886		
RSA (*n*)	1.098 (0.597, 2.019)	0.763		
DOR (*n*)	1.508 (0.981, 2.318)	0.061		
AMH (ng/ml)	1.055 (0.956, 1.165)	0.284		
Totaly gonadotropin dosage used (IU)	1 (1, 1)	0.749		
Estradiol level on trigger day (ng/L)	1 (1, 1)	0.532		
Number of aspirated oocytes (*n*)	0.981 (0.946, 1.018)	0.315		
Non PGT-A (*n*)	1.335 (0.87, 2.05)	0.186	1.943 (1.148, 3.289)	0.013*

*BMI* body mass index, *RSA* recurrent spontaneous abortion, *DOR* diminished ovarian reserve, *AMH* Anti-Mullerian hormone, *PGT-A* preimplantation genetic testing-aneuploidy. *GLMM* generalized linear mixed model, *OR* odds ratio, *CI* confidence interval. *p < 0.05

In the univariable analysis, female age, DOR, and PGT-A variables had a significant effect on live birth (Table 5). Variables with a significant effect in univariable evaluations were included in multivariable analysis as independent variables. In the GLMM, female age and PGT-A variables were found to be significant. When only age is taken into account, in cases under the age of 35, the probabilities of clinical pregnancy and live birth were found to be 5.548 and 5.850 fold higher than in the cases over the age of 43. In cases between the ages of 35–38, these probabilities were found to be 4.497 and 5.181 fold higher compared to the cases over the age of 43, respectively.

**Table 5:** Results of univariate and multivariate factors in relation to the live birth

Live birth	Univariable		Multivariable	
	*OR* (95% *CI*)	*P*-value	*OR* (95% *CI*)	*P*-value
Age group (years)		< 0.001*		< 0.001*
< 35	5.409 (1.884, 15.532)	0.002*	5.850 (1.979, 17.287)	0.001*
35–38	5.598 (1.926, 16.271)	0.002*	5.181 (1.742, 15.409)	0.003*
39–40	3.376 (1.119, 10.192)	0.031*	2.39 (0.769, 7.43)	0.132
41–43	2.165 (0.697, 6.725)	0.181	1.701 (0.532, 5.436)	0.370
> 43	Reference	-	Reference	-
BMI (kg/m2)	0.981 (0.954, 1.008)	0.170		
Duration of infertility (years, *n*)	0.986 (0.959, 1.015)	0.337		
RSA (*n*)	0.908 (0.593, 1.391)	0.658		
DOR (*n*)	0.734 (0.554, 0.973)	0.032*	0.851 (0.625, 1.159)	0.305
AMH (ng/ml)	1.033 (0.955, 1.118)	0.417		
Totaly gonadotropin dosage used (IU)	1 (1, 1)	0.492		
Estradiol level on trigger day (ng/L)	1 (1, 1)	0.363		
Number of aspirated oocytes (*n*)	1.018 (0.994, 1.042)	0.144		
PGT-A (*n*)	2.473 (1.812, 3.376)	< 0.001*	3.448 (2.43, 4.893)	< 0.001*

*BMI* body mass index, *RSA* recurrent spontaneous abortion, *DOR* diminished ovarian reserve, *AMH* Anti-Mullerian hormone, *PGT-A* preimplantation genetic testing-aneuploidy. *GLMM* generalized linear mixed model, *OR* odds ratio, *CI* confidence interval. *p < 0.05

## Discussion

The question of whether or not to perform a biopsy when only one embryo is available can pose a dilemma. On the one hand, unless additional cycles can be carried out, which is often not feasible, a single blastocyst may be the only chance the patient has of achieving pregnancy, and even the very small risk of damage to the embryo during biopsy creates anxiety. On the other hand, transferring the embryo blindly increases the risks of spontaneous abortion or chromosomal syndromes in the newborn. Recurrent miscarriages, the termination of an ongoing aneuploid pregnancy, or repeated implantation failures are extremely distressing for couples and may even deter them from further ART attempts. In addition, curettage and termination of an ongoing pregnancy may cause physical damage to the uterus and endometrium, which may put future transfer attempts at risk of failure. Morin et al. (2018) suggest that the true benefit of PGT-A in these patients is the avoidance of futile transfers and associated loss of time and the emotional burden of miscarriages and ongoing aneuploid pregnancies [15]. Therefore, our study was undertaken to evaluate whether PGT-A is beneficial for patients who have only one blastocyst available for biopsy.

Most prospective trials of PGT-A have included patients with normal ovarian reserve and favorable response to controlled ovarian stimulation. Ata et al. (2012) and Kahraman et al. (2016) showed that the probability of finding at least one euploid embryo significantly increased with each additional oocyte retrieved [9, 10]. Thus, the higher the patient’s ovarian reserve, the higher the chance of finding at least one euploid embryo. However, is PGT-A a good option for patients with low ovarian reserve who do not have multiple embryos for biopsy? There is a lack of data regarding the effectiveness of PGT-A in DOR patients. Debates still continue regarding which patients benefit most from PGT-A. Data have demonstrated that decreased miscarriage rates and reduced time to ongoing pregnancy were achieved when aneuploidy screening was performed. [5, 15]. Deng et al. (2020) with a limited patient number also found that PGT-A was associated with a lower miscarriage rate but concluded that it did not improve the live birth rate in poor ovarian reserve patients. However, in their study, one to four fresh or frozen unknown ploidy embryos were transferred on day 3 or 5 in a control group of non-PGT-A cases, whereas in the PGT-A group in each frozen embryo transfer cycle, single euploid embryo was transferred [16]. Thus, from this data, it is difficult to draw a firm conclusion regarding the efficacy of PGT-A for a patient with only single blastocyst available for transfer.

Comparison of pregnancy outcomes of patients with a single blastocyst who underwent embryo transfer with and without PGT-A was based on per embryo transfer. Calculating the cycle outcome according to per initiated cycle in this risky PGT-A group with a mean age of 38.6 years and DOR would underestimate the important role of PGT in reducing the number of futile transfers which would otherwise result in a miscarriage or a fetal aneuploidy or implantation failure. If per initiated cycle was taken into account, because 80% of cases had no embryo transfer, the cycle outcomes would be extremely low compared to the non-PGT-A group. However, these figures show that 80% of the embryos were not suitable for transfer and thus PGT-A prevented futile transfers, the emotional burden of miscarriages and ongoing aneuploid pregnancies and the risk of physical damage to the uterus and endometrium. The clinical pregnancy and live birth rate were significantly higher in the PGT-A group compared to the non-PGT-A group. Although the total pregnancy loss was found to be lower in the PGT-A, this was not a statistically significant difference. This could be explained by the fact that in the PGT-A group, as there was only one available euploid embryo for transfer, some moderate quality embryos and some delayed re-expanded embryos were transferred.

Mosaic embryo transfers were excluded from this study, firstly because in 21.7% of cases, aCGH was used, which may not detect mosaicism. Secondly, precise matching of mosaicism in both PGT-A and non-PGT-A groups would not have been feasible since the percentage of mosaicism and the number of chromosomes involved would vary widely in their incidence and effect on pregnancy outcomes.

Although the vast majority of our patients in this study fell into the DOR group as defined by Bologna ESHRE Criteria, in the PGT-A group, the number of patients with a diagnosis of advanced maternal age and/or DOR was significantly higher than in the non-PGT-A group. This can explain why the number of patients diagnosed with unexplained infertility was significantly lower in the PGT-A group compared to the non-PGT-A group. These patients had a higher risk of recurrent miscarriages, the termination of an ongoing aneuploid pregnancy, or repeated implantation failures and therefore, there was a strong patient preference for PGT-A. On the other hand, the majority of the non-PGT-A group also had DOR and only one embryo available for transfer, but within this younger group, there was a strong preference for fresh transfer. Thus, it was possible to compare two groups, non-PGT-A and PGT-A groups with a single embryo, but not to compare two groups with frozen transfers only, as would have been ideal.

The results of the GLMM were used for univariate and multivariate evaluations of risk factors affecting clinical pregnancy, total pregnancy losses, and live birth. GLMM results showed that PGT-A and female age were significant variables on clinical pregnancy, total pregnancy losses, and live birth and that PGT-A had a positive effect on pregnancy outcomes independent of all other variables. PGT-A in the presence of a single blastocyst significantly increases the chance of clinical pregnancy and live birth and decreases total pregnancy losses regardless of age. Female age also had a significant effect on pregnancy outcomes independent of all other variables. Patients under 35 years of age had a significantly higher probability of clinical pregnancy and live birth. In non-PGT-A cases, the probability of a total pregnancy loss was found to be almost twofold higher.

Our study, providing the results of a large number of PGT-A cases, addresses the dilemma in clinical practice of whether or not to perform a biopsy when only one embryo is available. Particularly with high-risk groups, this is a crucial question in clinical practice, with different options available: either the blind transfer of a blastocyst or embryo biopsy which can result the cancelation of the transfer, even in cases where patients may no longer produce more or even one oocyte in another cycle. Our results showed the significant benefit of PGT-A for clinical outcome, despite the fact that in only 20% of PGT-A cycles was a transferable, chromosomally normal embryo found.

## Conclusion

PGT-A in the presence of a single blastocyst significantly increases clinical pregnancy and live birth rates and decreases total pregnancy losses regardless of age. In addition, aneuploid embryo transfer cancelations prevent ineffective and potentially risky transfers.
